# Inverse-Designed Broadband All-Dielectric Electromagnetic Metadevices

**DOI:** 10.1038/s41598-018-19796-y

**Published:** 2018-01-22

**Authors:** F. Callewaert, V. Velev, P. Kumar, A. V. Sahakian, K. Aydin

**Affiliations:** 1 Department of Electrical Engineering and Computer Science, Northwestern University, Evanston, IL 60208 USA; 2 Department of Physics and Astronomy, Northwestern University, Evanston, IL 60208 USA

## Abstract

This paper presents a platform combining an inverse electromagnetic design computational method with additive manufacturing to design and fabricate all-dielectric metadevices. As opposed to conventional flat metasurface-based devices that are composed of resonant building blocks resulting in narrow band operation, the proposed design approach creates non-resonant, broadband (Δ*λ*/λ up to >50%) metadevices based on low-index dielectric materials. High-efficiency (transmission >60%), thin (≤2λ) metadevices capable of polarization splitting, beam bending, and focusing are proposed. Experimental demonstrations are performed at millimeter-wave frequencies using 3D-printed devices. The proposed platform can be readily applied to the design and fabrication of electromagnetic and photonic metadevices spanning microwave to optical frequencies.

## Introduction

Conventional optical elements that control the polarization, phase and amplitude of electromagnetic (EM) radiation such as lenses, polarizers, beamsplitters, and mirrors are typically engineered at a scale much larger than the wavelength. Within the last two decades, significant amount of research has been devoted to understand light-matter interactions and design novel materials and electromagnetic devices with subwavelength features. Metamaterials, and more generally materials composed of nanostructures with subwavelength feature size, have emerged as a viable platform to manipulate electromagnetic radiation in unconventional manners^1,2^. In particular, photonic crystals^3^ and negative-index materials^4^ have been used to achieve sub-diffraction lensing^5–7^. More recently, metasurfaces^8–10^ have gained substantial interest due to their ability to perform optical functionalities such as lensing^11^, holograms^12,13^ and beam shaping^14^ within an extremely thin layer. Although the ability to control phase, amplitude and polarization using subwavelength-thick metasurfaces is a promising route towards building miniature optical devices, they suffer from several drawbacks prohibiting their potential in replacing conventional bulk optical elements. Initial metasurface designs utilized plasmonic metals that exhibit high optical losses and thus were of relatively low efficiency^15^. Lossy metals have been replaced with high-index dielectric materials like amorphous silicon^16^, but such metasurfaces often rely on Mie-type resonances that result in a narrow wavelength range of operation^14,17^.

Typical metasurface design starts with identification of an optical resonator with a well-defined geometrical shape, such as triangles^18^, rectangles^12,19^, ellipses^14^ or V-antennas^8,15^. Phase information is then calculated for various geometrical parameters such as radius, width, orientation, etc.^16^. The number of degrees of freedom in the design of these shapes is very limited, which makes it difficult to optimize both efficiency and bandwidth of metadevices while achieving full control of the polarization. Here, we use an inverse electromagnetic method^20–26^ to design high-efficiency (>60%), broadband (Δλ/λ > 25%), dielectric-based thin (≤2λ) electromagnetic metadevices overcoming the aforementioned limitations. Inverse design opens up the entire design space to enable metadevices with increased and enhanced functionalities. In order to demonstrate the feasibility of our inverse design approach, we use additive manufacturing to print a low-loss polymer into a complex geometrical pattern. We demonstrate the design, fabrication and characterization of wavelength-scale metadevices for bending, polarization splitting and focusing of EM radiation at millimeter-wave frequencies.

## Design method

**Figure 1:**
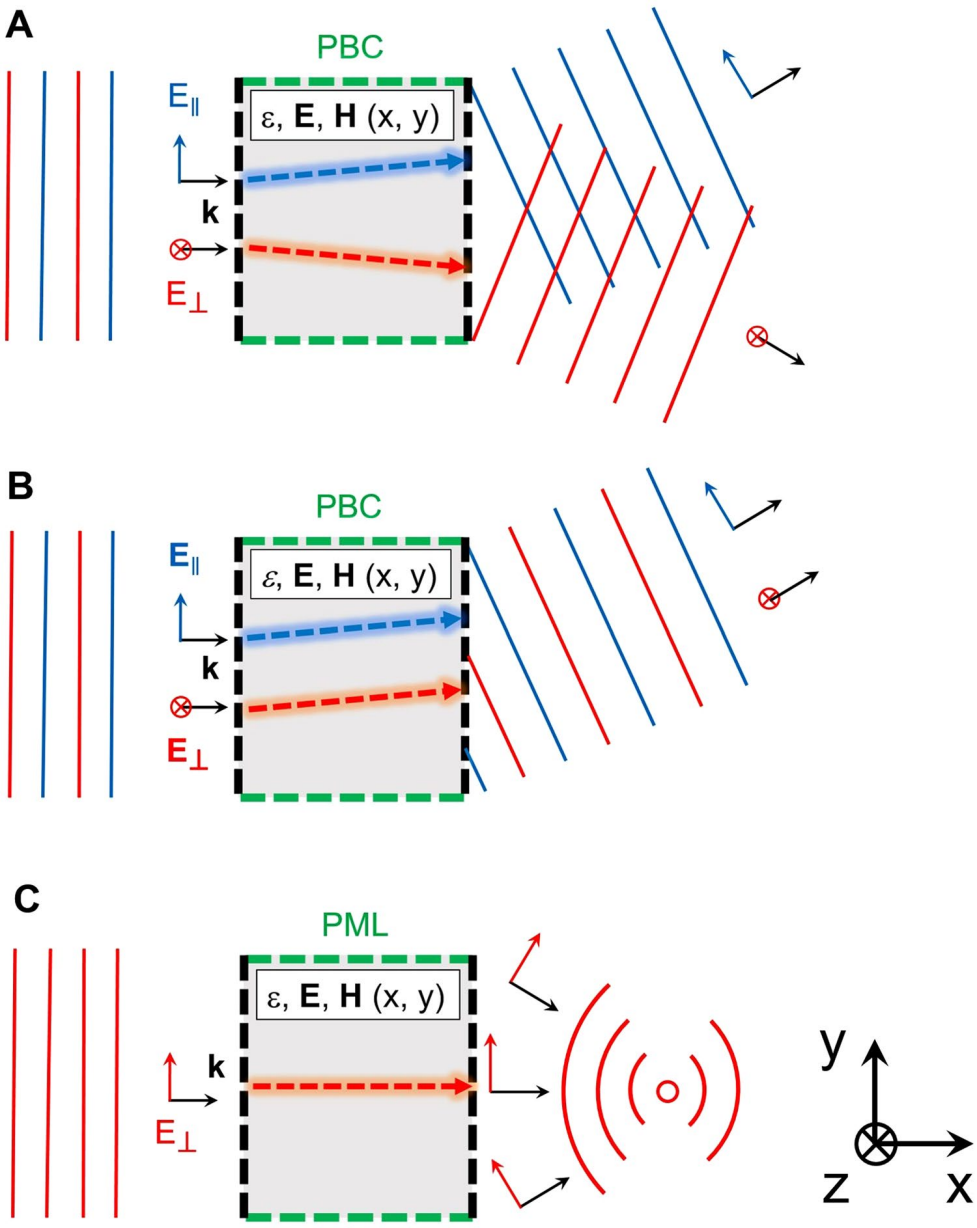
Schematics for the inverse electromagnetic approach for designing free-space metadevices. The desired optical functionality is defined by a set of input and output conditions at the boundaries of the design space. A polarization splitter (**A**) is a grating that converts normally incident plane waves of parallel and perpendicular polarizations into two different diffraction orders. A bending device (**B**) converts a normally incident plane wave into the same diffraction order. A flat metalens (**C**) is a device that converts a plane wave into a cylindrical wave converging to a chosen focal point.

First, we design two meta-gratings to behave as polarization beamsplitters for normally incident free-space radiation. As illustrated in Fig. 1A, those devices bend parallel and perpendicular polarizations to opposite diffraction orders. We also design a meta-grating that bends both polarizations to the same diffraction order (Fig. 1B). Finally, we propose a ~*λ*-thick flat metalens (Fig. 1C) that converts a normally incident plane-wave into a focusing cylindrical wave.

We use the objective-first algorithm^22,27,28^ in which the electromagnetic wave equation:

1 $${{\rm{\min }}}_{\varepsilon ,E}\nabla \times \nabla \times E-{w}^{2}\varepsilon E$$

is treated as an optimization problem. This nonlinear optimization is decomposed in two subproblems, where each variable (*ε* and **E**) is fixed while linear optimization is performed on the other variable, alternatively and iteratively, and where boundary electromagnetic field distributions reprenting the desired behavior are constraints of the model. There is no general method to find the optimum solution of (1), but the proposed method converges most of the times towards solutions that satisfy desired functionality with acceptable performance. An on-chip wavelength splitter^22^ and an optical diode^29^ have been successfully demonstrated using such inverse electromagnetic design approach.

Bending and polarization splitting are achieved using meta-gratings that convert an input plane wave to an output plane wave with a different diffraction order than *m* = 0, with periodic boundary conditions along the *x*-axis. For metalenses, we aim to focus an input plane wave at a desired focal distance; hence, the output is chosen to be the field of a cylindrical wave centered in the desired focal point, following the equation (approximation at large r):

2 $${E}_{z}(r)={E}_{z0}{r}^{-\frac{1}{2}}\exp \,ikr$$

where r is the distance from the focal point. Metalenses do not perform like a grating; therefore, we set the boundary conditions of a perfectly matched layer (PML) along the *x* direction. The designs are two-dimensional, which corresponds to metadevices with infinite height along *z*. In practice, the fabricated devices are ≈10*λ* thick.

## Experimental Section

### Design and fabrication

Inverse-designed metadevices are fabricated using additive manufacturing, commonly called 3D-printing. This bottom-up approach allows the fabrication of very complex devices with a large aspect ratio. Furthermore, 3D-printing is an incredibly scalable method, with resolutions ranging from 100 nm to 1 mm^30–32^, allowing the fabrication of electromagnetic devices for applications from the visible to the millimeter- wave and microwave regimes^33–36^. Here, we demonstrate the proposed devices in the millimeter-wave regime (*f* > 30 GHz) using high impact polystyrene (HIPS) and a consumer grade 3D-printer based on fused deposition modeling for the fabrication. The material is chosen for its low cost and very low attenuation in the microwave to millimeter-wave region, with a loss-tangent measured to be $\tan \,\delta  < 0.003$ over the 26–38 GHz band. In this band, the real part of the dielectric constant of HIPS $\varepsilon ^{\prime} \approx 2.3(n\approx 1.52)$, which is then used as a constraint in our algorithm to design binary devices made of air (*ε* = 1) and HIPS (*ε* = 2.3). Because of the low index, the phase difference between the input and output is approximately proportional to the effective thickness of the polymer. Therefore, in order to allow a 2*π* phase shift between a part full of polymer and a part full of air, the device thickness needs to obey:

3 $${\rm{\Delta }}\varphi =2\pi (n-1)\frac{t}{\lambda }=2\pi \times 0.52\times \frac{t}{\lambda }\ge 2\pi ,$$

which means that the minimum device thickness is around 2*λ*.

### Measurement setup

To test the electromagnetic properties of the devices, a vector network analyzer (VNA) generates the input signal that is transmitted through a high-gain horn antenna placed far away from the sample (distance > 100*λ*) in order to produce a plane wave perpendicularly incident on the input surface. The device is surrounded by radar absorbing material to prevent reflections from the surroundings. For the three meta-gratings, the transmitted power is measured in the far-field (>100*λ*) with a low-gain horn antenna as a function of the angle between −40° and 40° in 2° steps and as a function of the frequency between 26 GHz (11.5 mm) and 38 GHz (7.9 mm). For the lenses, the output power is mapped along the axial plane on the right side of the device using a probe antenna attached to a X-Y stage. The measurement starts around 1 cm to the right of the device due to technical limitations of the setup, which is the reason why the experimental intensity maps are truncated when compared to the simulated maps.

## Results

### Meta-gratings

**Figure 2:**
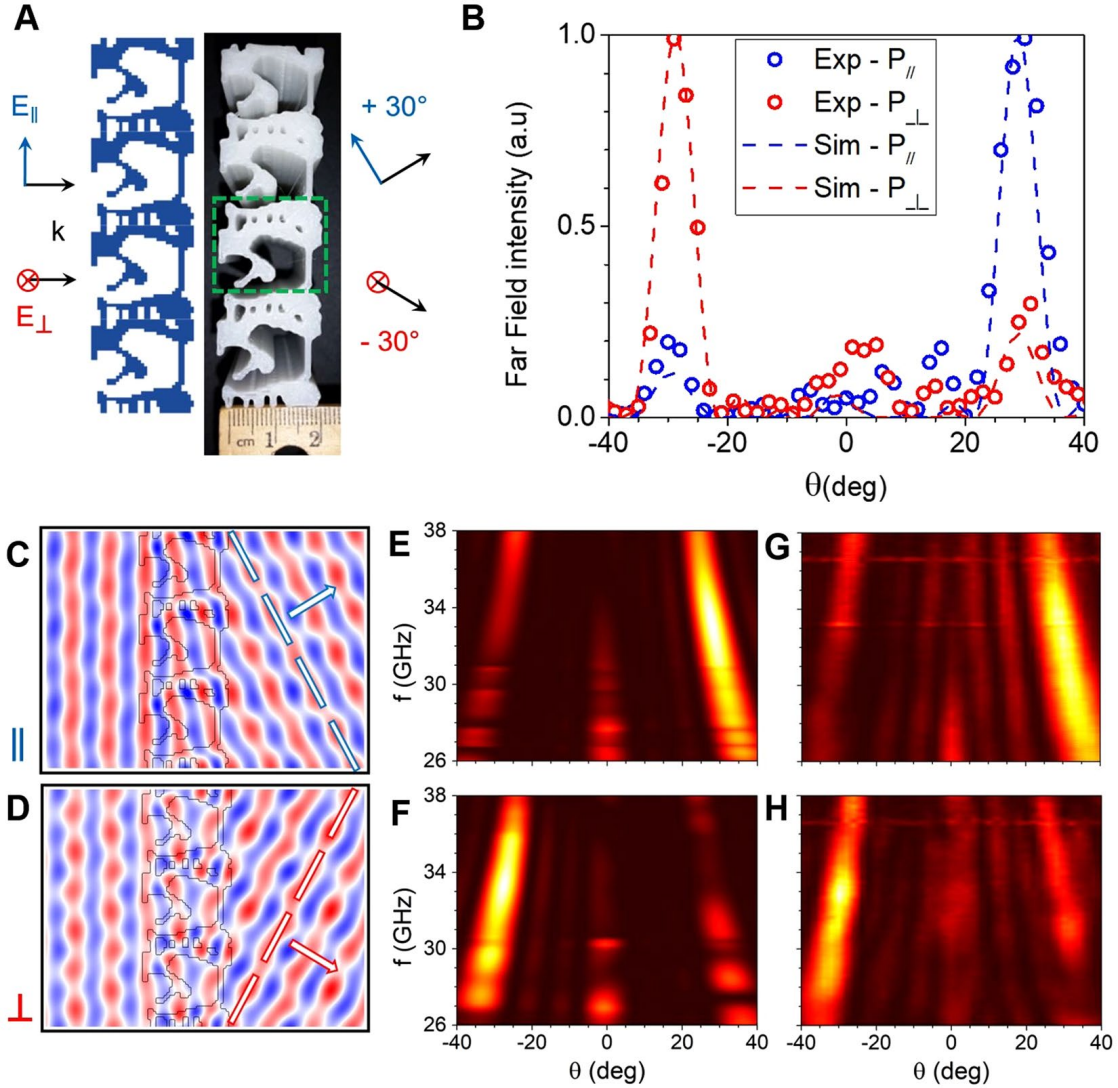
Inverse-designed polarization splitter. (**A**) Schematic drawing (left) and top-view photograph (right) of the 3D-printed 30*°* polarization splitter. The green rectangle indicates the unit cell of the grating. (**B**) Simulated (dashed lines) and measured (circles) far-field power as a function of deflection angle for both parallel and perpendicular polarizations. (**C**) and (**D**) Simulated **H**_*z*_ and **E**_*z*_ field amplitudes for parallel (**C**) and perpendicular (**D)** polarizations, respectively, at 33 GHz. (**E)** to (**H**) Simulated (**E**,**F**) and measured (**G**, **H**) far-field intensity profiles as a function of the output angle and the millimeter-wave frequency for parallel (**E**,**G**) and perpendicular (**F**,**H**) polarizations.

First, we demonstrate a free-space polarization splitter. The proposed metadevice (Fig. 2A) deflects a normal incident plane-wave polarized along *y* (parallel) and *z* (perpendicular) directions into *m* = +1 and *m* = −1 diffraction orders respectively with high efficiency and over a broad bandwidth. The width is chosen to be ~2*λ* to reach desired phase change as explained in the experimental section. The periodicity, *L* along *y* is determined by the deflection angle *θ* of the desired diffraction order *m* (here *m* = ±1 for all devices), following the grating equation $L\,\sin \,\theta =m{\lambda }$. We designed and optimized the metadevice for an operation frequency of 33 GHz (free space wavelength of *λ* = 9.1 mm) and a deflection angle of *θ* = ±30°, for which *L* = 1.8 cm. The inverse-design algorithm generates a binary refractive-index distribution of dielectric and air that is then printed with dimensions of 2 cm × 7.2 cm × 8 cm. A photograph of the 3D-printed metadevice is shown next to the computer-generated pattern in Fig. 2A, which shows the high fidelity of the 3D-printing method.

We measured the far-field angular transmission through the fabricated metadevice to verify the predicted polarization splitting behavior. Figure 2B plots the simulated and measured power distributions at 33 GHz. We observe that a plane wave with parallel polarization is bent at an angle *θ* = +30°, whereas the perpendicular polarization is deflected with an angle of *θ* = −30°. The total power transmitted by the metadevice at 33 GHz is measured to be 76% for the parallel polarization and 54% for the perpendicular polarization, which are lower than the simulated values of 90%. The discrepancy is likely due to structure imperfections in the fabricated devices, the finite number of periods in the printed structures as well as an imperfect plane-wave input. The rejection ratio, defined as the ratio between the peak intensity and the maximum intensity outside the main peak, is experimentally found to be 5.2 dB and 7.0 dB for the parallel and perpendicular polarizations, respectively, which are close to the simulated values of 6.6 dB and 9.3 dB, respectively.

We perform full-field electromagnetic simulations to calculate the electromagnetic properties of the metadevice. We plot the vertical fields, i.e. **H**_*z*_ for parallel polarization (Fig. 2C) and **E**_*z*_ for perpendicular polarization (Fig. 2D), at 33 GHz. The spatial field distribution provides a clear picture of how the EM waves propagate inside the metadevice. In metasurfaces based on resonant geometric elements, the phase change is due to the interaction of the incoming plane-wave to a strong Mie resonance mode^14^, which typically results in a relatively narrowband operation. Here, the phase change does not stem from the interaction with a specific resonant mode, but rather due to light propagation inside the dielectric structure, with a larger phase shift of 6π in a part filled with dielectric (*ε* = 2.3) than a 4π shift in a part mostly void (*ε* = 1.0). The polarization splitting is a result of the different phase-change response of the device to different polarizations owing to its complex dielectric shape.

Although we choose 33 GHz to be the frequency to optimize for highest efficiency in our inverse-design algorithm, we observe broad operation bandwidth that spans a range of frequencies from 27 to 38 GHz, for a relative bandwidth Δ*λ*/λ ≈ 33%, which is enabled by the inverse-design method favoring non-resonant dielectric structures. Figure 2 plots simulated (E,F) and measured (G,H) power transmitted in the far-field as a function of the angle and the frequency for parallel (E,G) and perpendicular (F,H) polarizations. The simulations and measurements agree relatively well.

**Figure 3:**
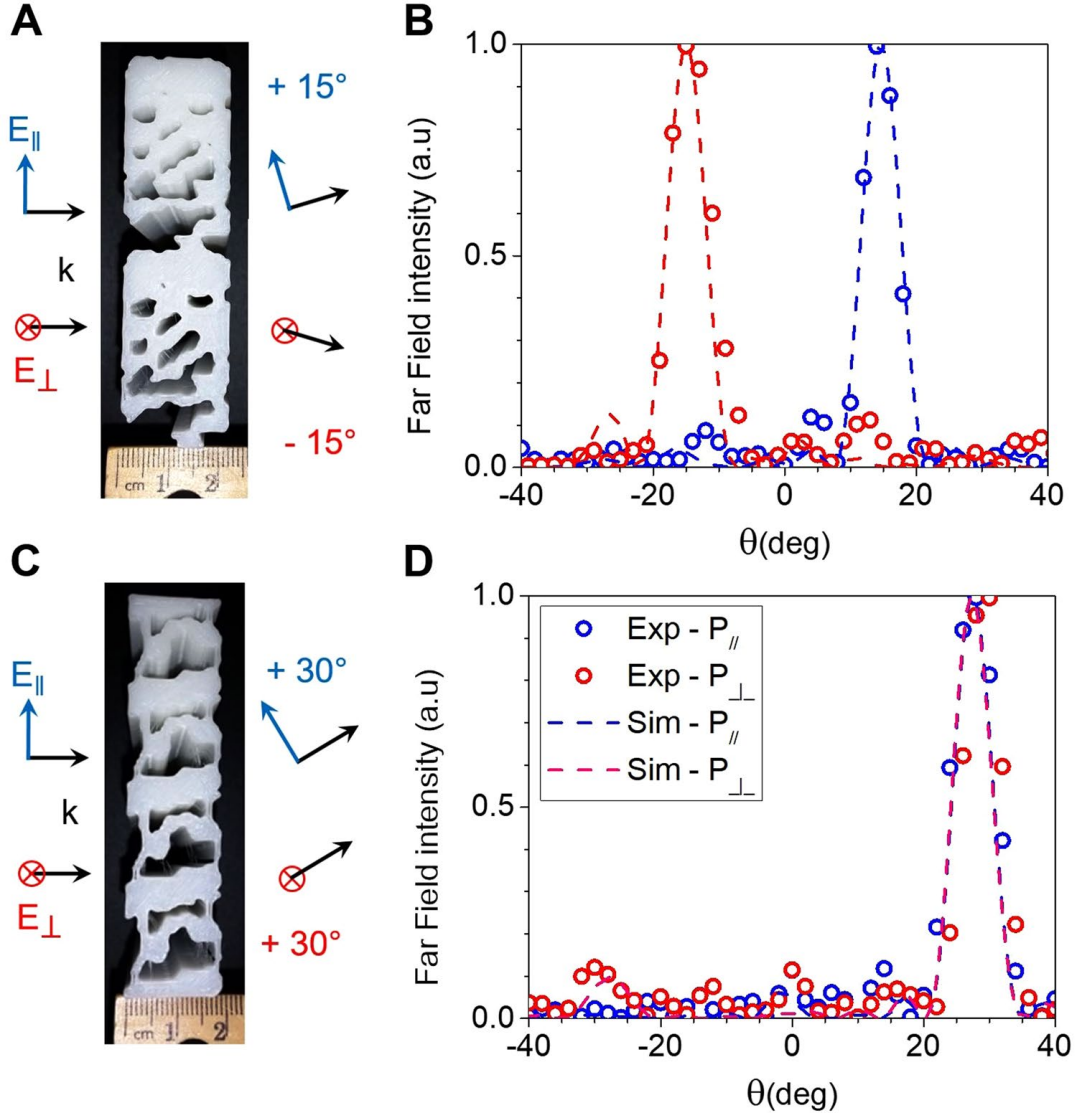
Inverse-designed meta-gratings. Photographs (**A**, and **C**) and simulated (dashed lines) and experimental (circles) far-field intensity plots of the 15*°* polarization splitter (**B**) and the 30*°* bending device (**D**) as a function of the output angle for a frequency of 33 GHz.

In order to demonstrate the versatility and flexibility of the inverse-design approach, we designed and fabricated two additional metadevices that bend the millimeter-waves. The first one is a polarization splitter with a bending angle of +/−15° (Fig. 3A). Similar to the 30° splitter, this device presents a gradient of dielectric filling fraction along the *y*-direction with a larger periodicity (*L* = 3.5 cm) in order to favor a smaller bending angle. The simulated and measured angular far-field transmitted powers are plotted for both polarizations at 33 GHz in Fig. 3B. The measured rejection ratios for the 15° splitter are 8.2 dB and 10.6 dB for parallel and perpendicular polarizations respectively. The device has a dielectric filling fraction with a similar profile to that of a bending device towards the +15° diffraction order. The polarization splitting is due to the coupling of perpendicular polarization to a resonant wave propagating along the *y*-direction inside the device, which reverses the bending direction. This explains the lower bandwidth of the device for perpendicular polarization, from 31 to 37 GHz (a relative bandwidth of 18%), compared to a very large bandwidth for the bending behavior of parallel polarization, from 22 to 44 GHz (data not shown). The designs, simulated fields and broadband far-field data are shown in Figure S1 in the supplementary information.

**Figure 4:**
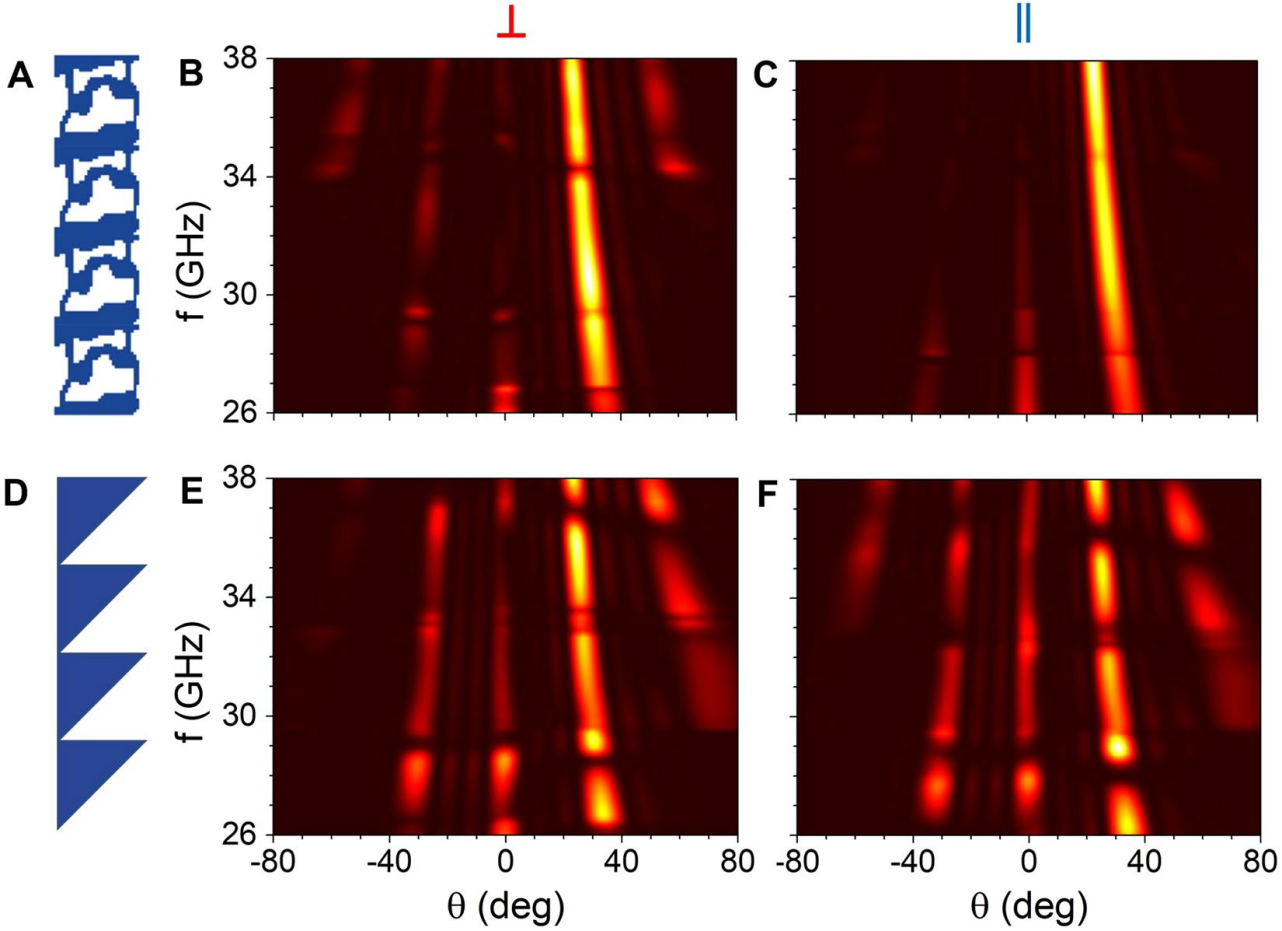
Comparison between the performance of the inverse-designed device (**A** to **C**) and a blazed grating (**D** to **F**) optimized to bend electromagnetic radiation by 30° independently of the polarization. The simulated far-field intensities are represented for angles from −80° to 80° and for frequencies from 26 GHz to 38 GHz for perpendicular (**B**,**E**) and parallel (**C**,**F**) polarizations. As can be seen, the inverse-designed metadevice transmits a much lower power to undesired grating orders (23% for perpendicular polarization and 18% for parallel polarization) than the blazed grating (47% for perpendicular polarization and 51% for parallel polarization). Simulated rejection ratios at 33 GHz are 10.1 dB and 12.4 dB for the inverse-designed bending device, compared to 6.6 dB and 3.8 dB for the triangular grating for perpendicular and parallel polarizations respectively.

In addition to polarization beam-splitter, we also designed and realized a polarization-independent bending device (Fig. 3C) which bends both polarizations to the same diffraction order. Simulations and experimental results of the far-field power at 33 GHz are shown in Fig. 3D, showing very good agreement between the theory and experiment. The designs, simulated fields and broadband far-field data are shown in Figure S2. Although polarization-independent bending of EM radiation can be achieved with a triangular blazed grating (Fig. 4D), such gratings deflect significant amount of power to higher diffraction orders, as shown in Fig. 4E,F. On average from 26 to 38 GHz, the inverse-designed device reduces the amount of power sent into undesired diffraction orders by a factor of 2.8 for parallel polarization and 2.0 for perpendicular polarization when compared to a blazed grating of similar thickness, which can be seen on the far-field power in Fig. 4B,C. The bending to the first diffraction order extends to 44 GHz with high efficiency (data not shown), which corresponds to a relative bandwidth of 55%, but significant power is diffracted to higher orders for larger frequencies.

### Metalenses

**Figure 5:**
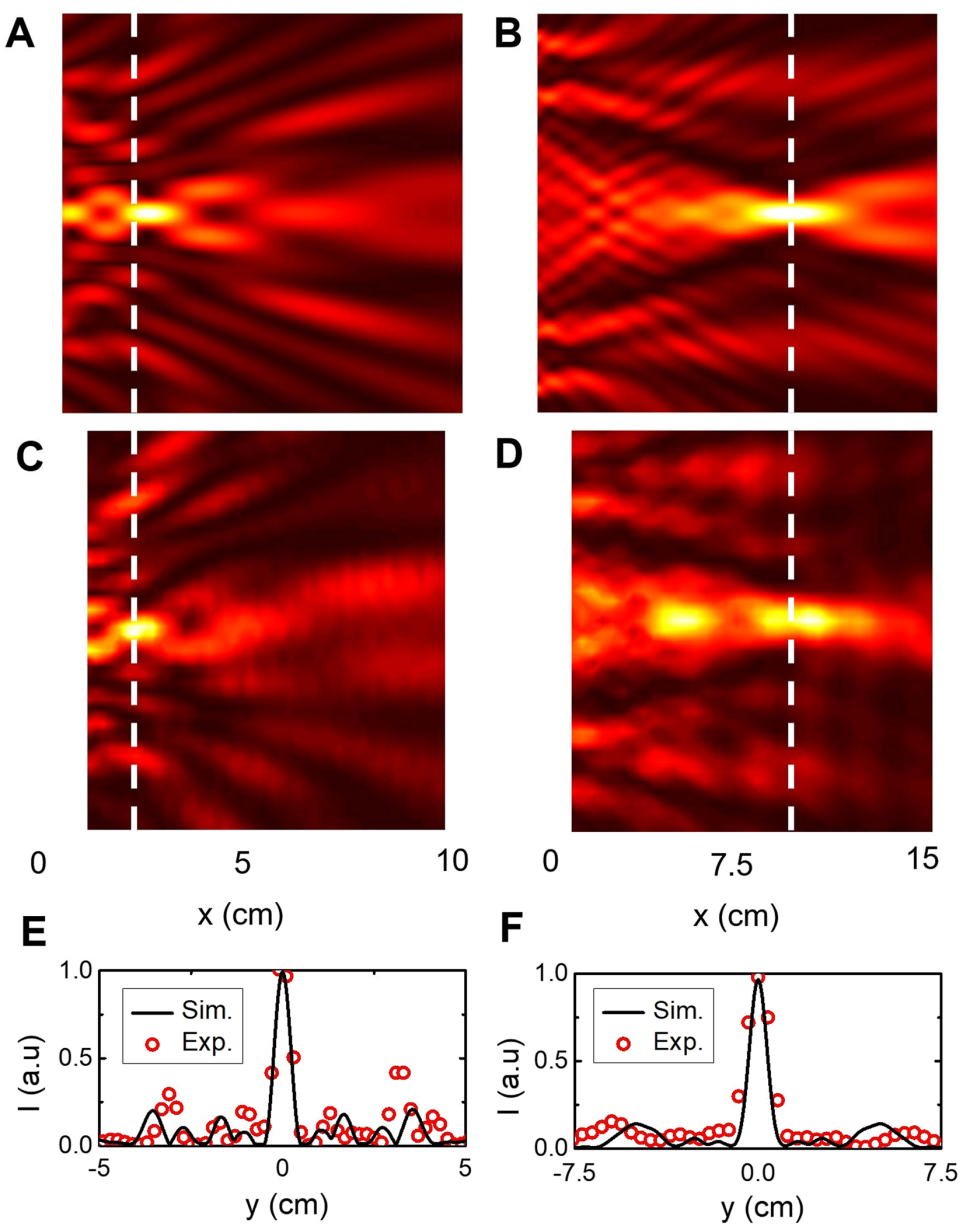
Inverse-designed metalenses. Simulated (**A**,**B**) and measured (**C**,**D**) spatial power distributions along the x-y *plane at* the output of the metalenses at 38 GHz. The input plane wave is generated by a horn antenna 1 m away on the left of the device while the output is measured with a probe antenna scanned along a 9 × 10 cm x-y plane for the first lens (**A**,**C**) and a 14 × 15 cm plane for the second lens (**B**,**D**). The first lens focuses perpendicularly polarized EM field 2λ away from the device whereas the second lens focuses it 15λ away. Schematics and pictures of the 3D-printed lenses are shown next to the simulated and experimental maps respectively. (**E**) and (**F**) Cross-section of the simulated (black line) and measured (red circles) power along the white dashed lines on the color maps for the first (**E**) and second (**F**) lens.

Next, we propose and design flat metalenses using the inverse-design algorithm and boundary conditions as illustrated in Fig. 1B. We designed and fabricated two different metalenses with focal lengths of 2*λ* and 15*λ*, respectively. Both lenses are optimized and scaled for operation around 38 GHz (*λ* = 7.9 mm). The first metalens is 1.5-cm wide, 10-cm long, the second is 2.5-cm wide and 15-cm long and both are 10-cm tall. A picture of each device is shown with the computer-generated design in Fig. 5.

The electromagnetic behavior of both devices is simulated with a perpendicularly-polarized incoming plane wave. The electric-field intensity profiles for the short-range and long-range metalenses are plotted in Fig. 5A,B, respectively. We also performed a 2D scan of the transmitted power behind the metalenses using a millimeter-wave probe antenna positioned at *z* = 5 cm. The measured spatial intensity distribution in the *x-y* plane for the short-range and long-range lenses are provided in Fig. 5C,D, respectively. The simulated and measured spatial-intensity distributions closely match. As expected, the first device focuses EM radiation 1.5 cm (~2λ) away from the device whereas the second device’s focal point is located 12 cm (~15λ) away. The full-width-at-half-maximum (FWHM) of the focused radiation for both devices are 0.5 cm and 1.1 cm as shown in Fig. 5E,F, respectively, corresponding to practical numerical apertures (NAs) of 0.8 and 0.36 respectively, close to the theoretical values of 0.82 and 0.53, respectively. The proposed devices also show broadband focusing behavior from 28 GHz to 40 GHz. We provide the measured and simulated intensity profiles for operation at 30 GHz in Figure S3.

## Conclusion

We have presented a platform combining an inverse electromagnetic design algorithm with additive manufacturing for the design and fabrication of novel millimeter-wave metadevices. The proposed methodology can be generalized to any photonic device where the desired electromagnetic behavior can be defined in terms of input and output field distributions. Although we design and demonstrate metadevices in the millimeter-wave region, due to the scalability of Maxwell’s equations, similar devices can be designed to operate in any wavelength from visible to microwave frequencies provided that low-loss dielectric materials can be additively fabricated with subwavelength feature sizes. The presented platform addresses the need for rapid versatile design and prototyping of compact, low-cost, low-loss, and broadband components that can then be easily integrated into complex electromagnetic systems.

### Data availability statement

No datasets were generated or analyzed during the current study.

## Electronic supplementary material

Supplementary Information
