# Supplementary material for: Inverse-Designed Broadband All-Dielectric Electromagnetic Metadevices
Source: Sci Rep. 2018 Jan 22;8:1358. doi: 10.1038/s41598-018-19796-y (PMC5778146; doi:10.1038/s41598-018-19796-y)
Supplement: Supplementary file 1 — Supplementary Information [file 41598_2018_19796_MOESM1_ESM.pdf]

Supplementary materials for:

Inverse-Designed Broadband All-Dielectric Electromagnetic Metadevices

F. Callewaert[1], V. Velev[2], P. Kumar[1,2], A.V. Sahakian[1], and K. Aydin[1,*]

.

[1] Department of Electrical Engineering and Computer Science, Northwestern University, Evanston, IL 60208, USA

[2] Department of Physics and Astronomy, Northwestern University, Evanston, IL 60208, USA

* Correspondence and requests for materials should be addressed to K. A. (aydin@northwestern.edu)

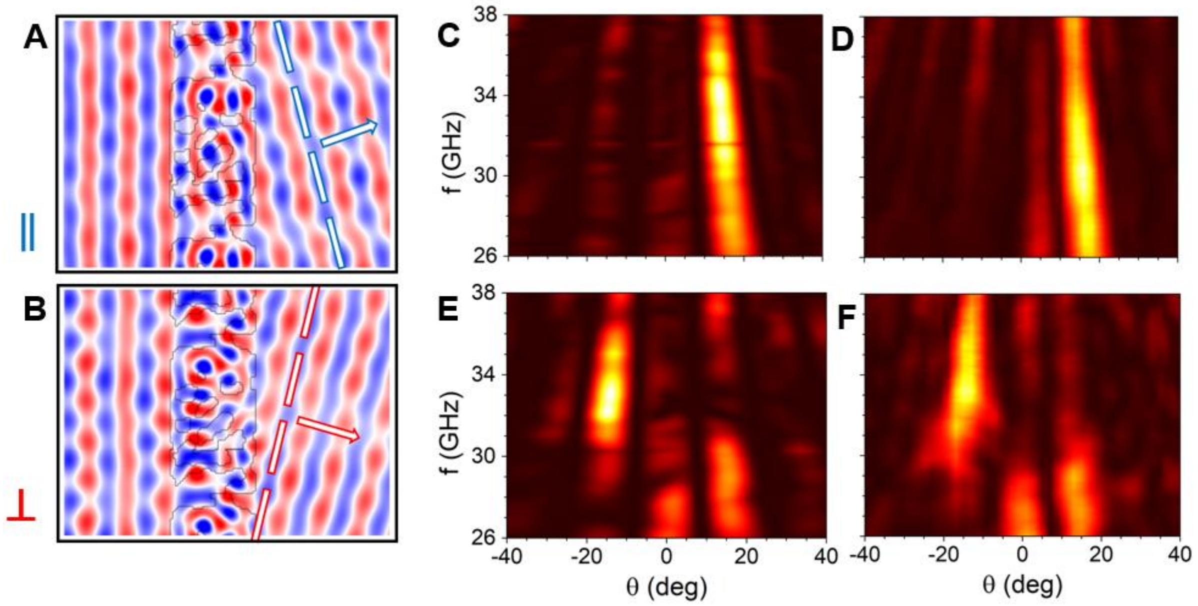

Figure S1. 15° polarization splitter. Simulated H_z (A) and E_z (B) fields in the 15° polarization splitter with a perpendicularly incoming plane wave for parallel (A) and perpendicular (B) polarizations and at a frequency of 33GHz. Simulated (C, E) and experimental (D, F) far-field intensity color maps as a function of the output angle between -40° and 40° and as a function of the frequency between 26GHz and 38GHz for both parallel (C, D) and perpendicular (E, F) polarizations.

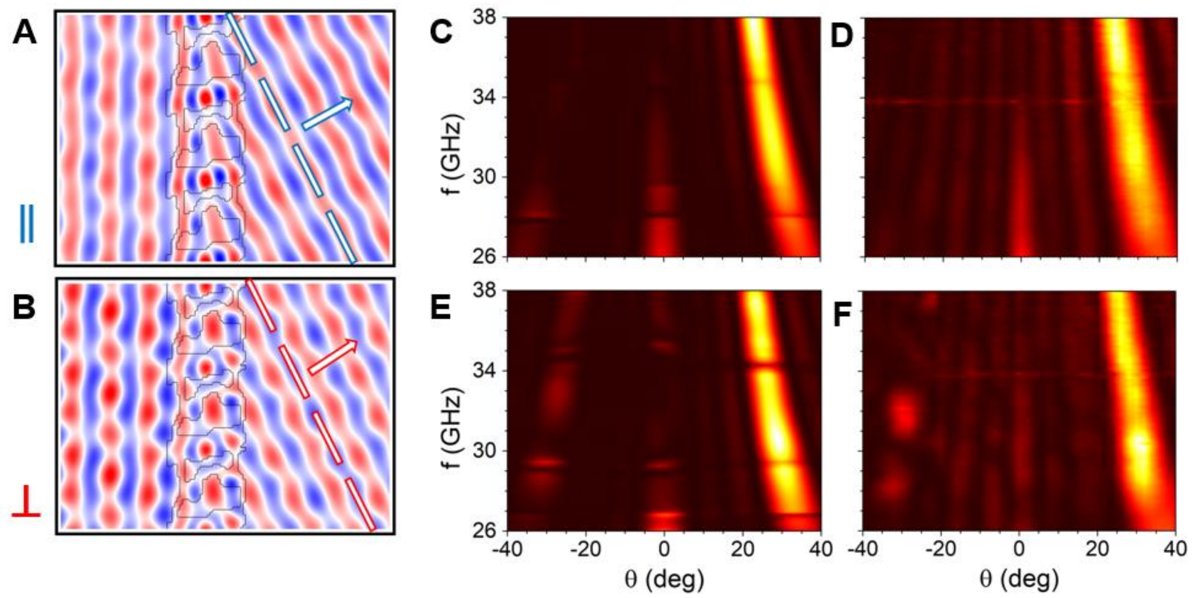

Figure S2. 30° bend. Simulated H_z (A) and E_z (B) fields in the 30° bending device with a perpendicularly incoming plane wave for parallel (A) and perpendicular (B) polarizations and at a frequency of 33GHz. Simulated (C, E) and experimental (D, F) far-field intensity color maps as a function of the output angle between -40° and 40° and as a function of the frequency between 26GHz and 38GHz for both parallel (C, D) and perpendicular (E, F) polarizations.

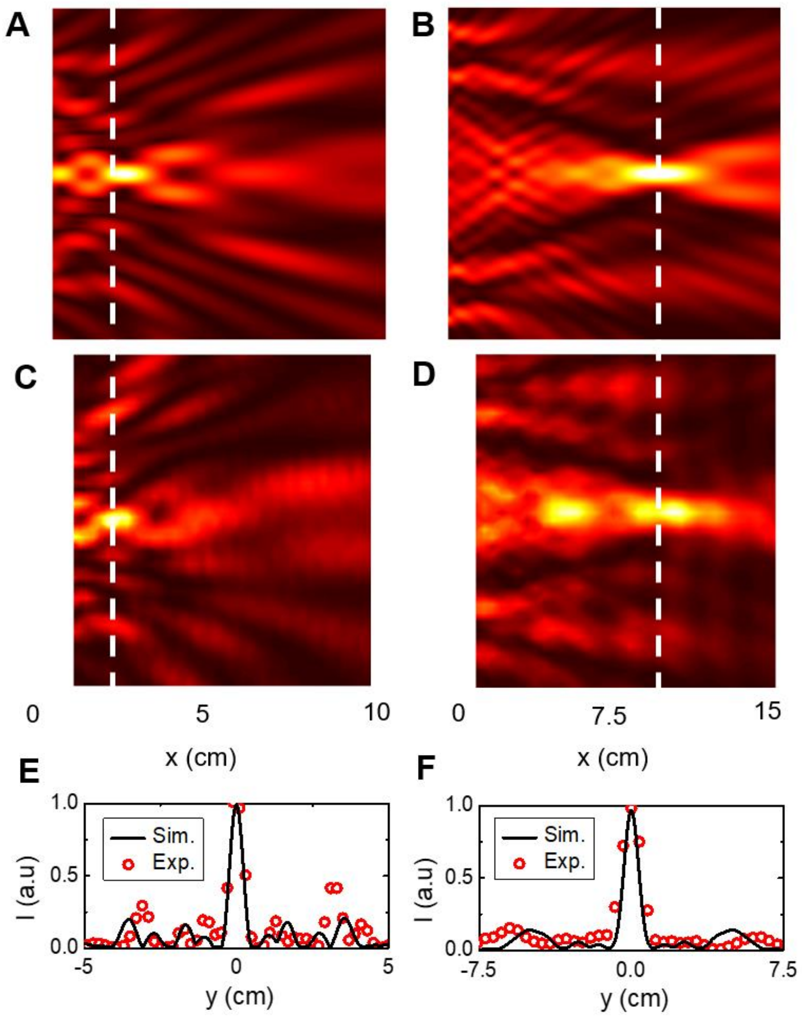

Figure S3. Inverse-designed metalenses at 30GHz. Simulated (A, B) and experimental (C, D) electromagnetic intensity color maps along the x-y plane at the output of the devices at a frequency of 30 GHz for the first (A, C) and second (B, D) lenses. (E) and (F): Cross-section of the simulated (black line) and measured (red circles) power along the white dashed lines on the color maps for both lenses.
